# Health-related quality of life in children with cystic fibrosis: validation of the German CFQ-R

**DOI:** 10.1186/1477-7525-7-97

**Published:** 2009-12-02

**Authors:** Anne Schmidt, Kerstin Wenninger, Nadja Niemann, Ulrich Wahn, Doris Staab

**Affiliations:** 1 Department of Paediatric Pulmonology and Immunology, Charité Universitätsmedizin Berlin, Campus Virchow Klinikum, Augustenburger Platz 1, 13353 Berlin, Germany; 2 Department of Paediatric Haematology-Oncology, University of Freiburg, Mathildenstrasse 1, 79106 Freiburg, Germany; 3 Centre for Quality Management in Health Care, Berliner Allee 20, 30175 Hannover, Germany

## Abstract

**Background:**

This study evaluates the psychometric properties of the Child and Parent versions of the German CFQ-R (Cystic Fibrosis Questionnaire Revised), a disease-specific measure of Health-Related Quality of Life (HRQoL) in children with cystic fibrosis (CF). Self-Rating is combined with proxy-rating by parents in the use of the questionnaire.

**Methods:**

136 children with CF (6 - 13 years) and their parents were recruited to evaluate internal consistency (Cronbach's α) and validity, 20 children and parents to examine reproducibility (ICC).

**Results:**

Cronbach's α is high in all but two dimensions of the Child version (α = 0.23-0.77) and for all dimensions of the Parent version (α = 0.69-0.89). For both questionnaires, reproducibility is moderate to high (ICC = 0.50-0.94). Factor analysis shows loadings of >0.4 in the majority of items. Higher HRQoL is reported by children with mild disease compared to those with moderate/severe disease and by boys compared to girls. Convergence between self-rating and proxy-rating depends on the dimension.

**Conclusion:**

The German CFQ-R, Child and Parent versions, are reliable and valid measures of HRQoL. They should be administered in combination as both, child and parent, provide important information. The measure offers a new patient-reported outcome for clinical purposes as well as for national and international studies in schoolchildren.

## Background

Cystic fibrosis (CF) is the most common hereditary life-shortening disease in the Caucasian population. Due to improved symptomatic therapies, survival periods have increased dramatically in recent decades; current median survival in Germany is observed to be 38.6 years in 2006 [1,2]. As a consequence, not only the quantity of years but also the quality of life has become more important. Numerous disease-related symptoms as well as the treatment burden have an impact on daily life [3].

There is evidence to suggest that surrogate parameters, such as the forced expiratory volume in one second (FEV_1_), do not correlate with patients' well-being [4]. Therefore, there is a growing need for such patient-reported outcomes (PROs) as Health-Related Quality of Life (HRQoL) [5]. The latter is defined as a multi-dimensional construct which allows awareness of a patient's subjective perception of the disease and the daily limitations they face [6]. There are various possible ways of assessing HRQoL. The importance of using disease-specific instruments especially in patients with CF has been described in detail [5,7-11].

There are three disease-specific instruments that are currently available for patients with CF; among them, the CFQ (Cystic Fibrosis Questionnaire) is the only one with age appropriate versions [11-13]. It was originally developed in France and then translated into several languages. Translation into German followed international recommendations and was described by Wenninger et al. [8,14].

The first German Child and Parent versions were tested in n = 44 children and their parents, but showed unsatisfactory psychometric properties (Wenninger, personal communication). The first US versions showed the same unsatisfactory psychometric properties. For this reason, a revision of the German questionnaires, including those for adults and adolescents, was carried out in cooperation with A. Quittner's group in the USA [8,11,15-17]. Revision involved the deletion and rewording of several items. The Revised CFQ, CFQ-R, showed good psychometric properties for the German versions for adolescents (CFQ-R 14+) and adults (CFQ-R 18+) [8]. The German CFQ-R versions are identical to the US versions of the CFQ-R [17] except for the language. Meanwhile, the versions for adolescents and adults are validated in a variety of languages, e.g. in French, German, English, Dutch and Danish, which enables researchers to undertake international multi-centre studies [8,11,15,16,18-21].

International studies on HRQoL in children are needed to gain a better understanding of the impact of public policies, interventions, therapies, and treatments [22]. Data on psychiatric problems in children with CF are heterogeneous, some studies indicate a higher incidence of psychiatric, psychosocial, emotional, and educational problems in children with CF than in their healthy peers [23,24]. In all age groups of patients with CF, depression shows higher rates than in healty populations, which might have a negative impact on adherence, family functioning and HRQoL [25]. In spite of the fact that measures for HRQoL are not able to record all impacts of a disease on daily life, they allow investigations in large populations.

Considering the advances in CF therapy and with regard to the recent increase in interest in PROs, the German CFQ-R, Child, and Parent versions are necessary tools for including schoolchildren in the analysis of HRQoL in patients with cystic fibrosis.

This study tests the psychometric properties of the revised German version for children aged 6 to 13 years (CFQk-R, here called "CFQ-R, Child version") and their parents (CFQe-R, here: "CFQ-R, Parent version"). It is hypothesized that the questionnaires are objective, reliable, and valid measures for HRQoL in schoolchildren with CF.

## Methods

### Participants and procedures

Children aged 6 to 13 years and their parents were recruited for the HRQoL survey during routine visits to the outpatient clinics of 10 German CF centres participating in the Benchmarking Project, which has been described in detail by Stern et al. [2]. The number of children of this age who participated in the Benchmarking Project in 2005 was 293, and 136 of these children (46%) could be included in this study. Only patients from one centre (n = 28) were accessible for test-retest reliability. Eight of them had to be excluded from the analysis due to the worsening of their condition after the first use of the questionnaire, leading to a final number of 20 patients in this subgroup. Approval was given by one local ethical committee (University of Tübingen) and accepted as representative of all participating CF centres. Age, sex, lung function (forced expiratory volume in one second, FEV_1_), nutritional state (weight for height, WH) and infection with Pseudomonas aeruginosa were assessed. The following inclusion criteria had to be met by the patients and parents: (1) diagnosis of CF either by positive sweat test or two identified CF mutations, (2) age 6 to 13 years, (3) ability to read and speak German, (4) assent of the child and written informed consent by the parents. Furthermore, patients for the evaluation of test-retest reliability had to remain in stable health and life conditions after the first survey for at least two weeks according to parents' and patients report. The questionnaires were used before the physician was consulted. In the test-retest sample, the second survey took place either at the patient's home or at the CF centre.

Participation rate was 136 out of 293 patients who were treated at the ten CF centres taking part in the Benchmarking Project. Children are characterized in table 1. The stable subgroup to determine test-retest reliability consists of 20 patients with comparable characteristics. Compared with the cohort of 293 patients aged between 6 and 13 treated at the participating centres during this period, the patients included in this study have a lower percentage of children with good lung function and nutritional state. Time elapsed from the communication of diagnosis to the test administration was more than one year in all patients.

**Table 1:** Characteristics of the patients

	Whole sample n = 136	Test-retest sample n = 20 (subgroup)	Benchmarking population n = 293
Age of the child in years (mean)	10.2 (SD 1.9) (range 6-13)	9.6 (SD 2.6) (range 6-13)	9.8 (SD 2.3) (range 6-13)
			
FEV_1 _(mean)	86% (SD 19) (range 31-125)	81% (SD 21) (range 41-109)	94% (SD 20) (range 31-159)
Percentage of children with FEV_1_>80%	68%	50%	80%
			
WH (mean)	97% (SD 12) (range 68-141)	94% (SD 9) (range 78-108)	99% (SD 12) (range 68-146)
Percentage of children with WH >90%	74%	65%	80%
			
Boys	55%	75%	49%
Mother completing CFQ-R-Parent	71%	85%	
Father completing CFQ-R-Parent	27%	15%	
Other person completing CFQ-R-Parent	1%	-	

FEV_1 _forced expiratory volume in one second in % predicted

WH weight for height in % predicted

Benchmarking population: Population of children with cystic fibrosis assessed in a project that was initiated to improve the quality of care for patients in Germany. The project was described by Stern et al. [2]

### Measures

#### The questionnaire

The German CFQ-R, Child version, is a self-rating questionnaire consisting of 35 items grouped into eight dimensions of HRQoL; the Parent version is a proxy-rating questionnaire with 43 items and eleven dimensions where parents report on their child's HRQoL. Some dimensions are exclusively represented in one of the two questionnaires. Table 2 shows details on dimensions, the number of items and representative items. All items are shown in additional files 1 and 2. The different scales correspond with five generic and three disease-specific dimensions of HRQoL, three symptom scales and a rating for "Subjective Health Perception". The questionnaire is self-administered by parents and by children between 11 and 13 years after checking for adequate reading skills; children between 6 and 10 years are interviewed and choose the answers from a special answering card. Items are in German and refer to a time frame of the preceding two weeks. In the Child version they are answered on a four-point Likert scale either on a true-false rating ranging from "not at all true" to "very true", or on a frequency response ranging from "never" to "often". In the Parent version, similar four-point Likert scales are used supplemented by five items with 4 eligible standard phrases for answer selection. Some items are recoded and then the score for quality of life is calculated on a scale between 0 (low) and 100 (high).

**Table 2:** The German CFQ-R, Child and Parent versions

	CFQ-R Child version	CFQ-R Parent version	Item examples for each dimension
Dimensions of Health-Related Quality of Life	(no. of items)	(no. of items)	
Physical Functioning	6	9	*"Child was able to run, jump and climb as it wanted."*
Energy	-	5	*"Child seemed energetic."*
Emotional State	8	5	*"Child felt worried."*
Social Limitations	7	-	*"Child felt left out."*
School Performance	-	3	*"Child has trouble concentrating."*
Body Image	3	3	*"Child felt physically different from others."*
Eating Disturbance	3	2	*"Child was pushed to eat."*
Treatment Burden	3	3	*"Child was bothered by undergoing the treatments."*
Subjective Health Perception	-	3	*"Child leads a normal life."*
Respiratory Symptoms	4	6	*"Child coughed during the day."*
Digestive Symptoms	1	3	*"Child's stomach hurt."*
Weight Problems	-	1	*"Child had trouble gaining weight."*

**No. of dimensions**	8	11	
**No. of items**	35	43	

#### Measures of health state

All data on health state are obtained by standardized procedures and reported to the German Registry database. Pulmonary function tests are performed to measure lung function. FEV_1 _> 80% is regarded as a good physical condition [2]. The established classification of disease severity differentiates between mild (FEV_1 _≥ 70%), moderate (FEV_1 _40-69%) and severe disease (FEV_1 _≤ 39%) [26,27]. Only one child with severe disease is included in this study.

Nutritional state is measured by weight for height. A value of ≥ 90% is regarded as good. Infection with Pseudomonas aeruginosa is assessed by throat swabs or sputum analysis [2].

### Statistical methods

Statistical analysis is conducted using the Statistical Package for Social Sciences (SPSS 13.0) with a level of significance of <0.05. For reliability, internal consistency is calculated using Cronbach's α (α>0.6 expected for all dimensions) [28,29]. The reproducibility of the results of the questionnaires is analysed by test-retest reliability using intra-class correlation (ICC). An ICC value of at least 0.6 for all dimensions is expected [30]. Construct validity is evaluated by factor analysis (Principal Component Analysis, PCA) to test the given structure of the CFQ-R. The number of factors is set at 8 for the Child version and at 11 for the Parent version. Correlation between the single item and its dimension is tested (item-internal consistency), with a recommended correlation of at least 0.4. Every item should be more closely correlated with its own dimension than with others (item-discriminant validity) [31,32].

Known-group validity is analysed using the T-Test. Comparisons are made between patients with mild and moderate/severe disease and between boys and girls. For all known-group comparisons, T-Tests and χ^2^-Tests for the detection of possible confounders are performed as post-hoc analyses.

The convergent validity of the Child and Parent versions is calculated by ICC. Here, ICC provides information about the degree of correlation between self- and proxy rating; high values indicate a higher level of correlation.

## Results

### Reliability

The data on n = 136 children are analysed for internal consistency (see table 3). For the CFQ-R, Child version, Cronbach's α is >0.6 in the dimensions "Physical Functioning", "Emotional State", "Eating Disturbance" and "Respiratory Symptoms". It is 0.57 in the "Body Image" dimension. Two dimensions ("Treatment Burden", "Social Limitations") have a low Cronbach's α and one dimension ("Digestive Symptoms") has only one item, so that α cannot be calculated. For the Parent version, the Cronbach's α values of all dimensions are >0.6. Again, α cannot be calculated for a dimension with only one item ("Weight Problems").

**Table 3:** Reliability of the German CFQ-R, Child and Parent versions

	CFQ-R Child version				CFQ-R Parent version			
**Dimensions of Health-Related Quality of Life**	**No. of items**	**Internal consistency** **(Cronbach's α)** **(n = 136)**	**Test-retest reliability** **(ICC)** **(n = 20)**	**95% Confidence Intervall for ICC**	**No. of items**	**Internal consistency (Cronbach's α)** **(n = 136)**	**Test-retest reliability (ICC)** **(n = 20)**	**95% Confidence Intervall for ICC**

Physical Functioning	6	0.77	0.80	0.57-0.92	9	0.89	0.87	0.71-0.95
Energy	-	-	-		5	0.69	0.90	0.76-0.96
Emotional State	8	0.69	0.74	0.47-0.89	5	0.66	0.86	0.69-0.94
Social Limitations	7	0.23	0.65	0.31-0.85	-	-	-	
School Performance	-	-	-		3	0.73	0.61	0.23-0.83
Body Image	3	0.57	0.83	0.63-0.93	3	0.82	0.94	0.85-0.98
Eating Disturbance	3	0.69	0.70	0.39-0.87	2	0.86	0.91	0.79-0.96
Treatment Burden	3	0.24	0.61	0.24-0.83	3	0.72	0.89	0.74-0.95
Subjective Health Perception	-	-	-		3	0.71	0.83	0.62-0.93
Respiratory Symptoms	4	0.64	0.83	0.63-0.93	6	0.83	0.89	0.75-0.96
Digestive Symptoms	1	-	0.50	0.09-0.76	3	0.69	0.83	0.63-0.93
Weight Problems	-	-	-		1	-	0.68	0.35-0.86

For test-retest reliability, a subgroup of 20 patients with stable health and life conditions completes the questionnaires twice (see table 3). The average time between the first and second use of the CFQ-R is 13.5 days (range 9-17). ICC is above the expected value of 0.6 for 7 of the 8 dimensions of the Child version, for "Digestive Symptoms" ICC shows a value of 0.5. In all dimensions of the Parent version, ICC values above 0.6 are found.

### Construct validity

Factor analysis shows the loading of each item on the provided factor of the questionnaires (see additional files 3 and 4). Most items show highest loadings on the factor they are assigned to, except for nine out of 35 items in the Child version and ten out of 43 items in the Parent version. Total variance explained by the factors is shown in the additional files 5 and 6.

### Known-group validity

#### Severity groups

Data on health state at the time of questionnaire administration is available in 112 of the 136 children included in the study. Since there is only one child with severe disease, only two disease severity groups are compared (mild disease, n = 88; and moderate/severe disease, n = 24). In both the proxy and the self-rating, children with mild disease show significantly higher scores in "Physical Functioning" and "Respiratory Symptoms". In the self-rating there is also a significantly higher HRQoL in "Body Image". The parents' rating shows a significant difference in "Subjective Health Perception", a dimension that is not represented in the self-rating (see table 4). Post-hoc analysis shows a similar boy-girl ratio in both groups. Children with mild disease have significantly better WH, less Pseudomonas aeruginosa infection, and are younger than children with moderate/severe health state.

**Table 4:** Known Group Validity of the German CFQ-R, Child and Parent versions: Severity groups - Comparison of mean scores by T-Test

	CFQ-R Child version			CFQ-R Parent version		
**Dimensions of Health-Related Quality of Life**	**Children with mild disease (n = 88)**	**Children with moderate/severe disease (n = 24)**	**Sign**.	**Children with mild disease (n = 88)**	**Children with moderate/severe disease (n = 24)**	**Sign**.

Physical Functioning	**79**	**67**	**.01**	**87**	**72**	**.002**
Energy	-	-		69	63	ns
Emotional State	78	80	ns	82	76	ns
Social Limitations	60	60	ns	-	-	
School Performance	-	-		75	67	ns
Body Image	**82**	**70**	**.02**	74	65	ns
Eating Disturbance	85	80	ns	75	65	ns
Treatment Burden	66	68	ns	58	52	ns
Subj. Health Perception	-	-		**77**	**65**	**.01**
Respiratory Symptoms	**78**	**68**	**.01**	**79**	**63**	**<.01**
Digestive Symptoms	74	75	ns	72	69	ns
Weight Problems	-	-		58	49	ns

#### Gender

Girls' (n = 61) and boys' (n = 75) HRQoL is compared. For detailed results see table 5. Significant differences can only be seen in the self-rating, but not in the proxy rating. Girls show significantly lower values in "Emotional State", "Treatment Burden" and "Digestive Symptoms". Again, information on health state is only available in 112 out of 136 children. No significant differences are found in the variables age, WH, FEV_1 _or Pseudomonas aeruginosa infection.

**Table 5:** Known Group Validity of the German CFQ-R, Child and Parent versions: Gender differences - Comparison of mean scores by T-Test

	CFQ-R Child version			CFQ-R Parent version		
**Dimensions of Health-Related Quality of Life**	**Female patients** **(n = 61)**	**Male patients** **(n = 75)**	**Sign**.	**Female patients** **(n = 61)**	**Male patients** **(n = 75)**	**Sign**.

Physical Functioning	73	87	ns	80	85	ns
Energy	-	-		67	66	ns
Emotional State	**74**	**80**	**.03**	81	80	ns
Social Limitations	62	59	ns	-	-	ns
School Performance	-	-		75	70	ns
Body Image	78	76	ns	75	67	ns
Eating Disturbance	86	82	ns	75	66	ns
Treatment Burden	**62**	**70**	**.03**	53	54	ns
Subj. Health Perception	-	-		72	74	ns
Respiratory Symptoms	74	76	ns	72	76	ns
Digestive Symptoms	**67**	**76**	**.03**	72	71	ns
Weight Problems	-	-		59	52	ns

### Convergent validity

**Figure 1:**
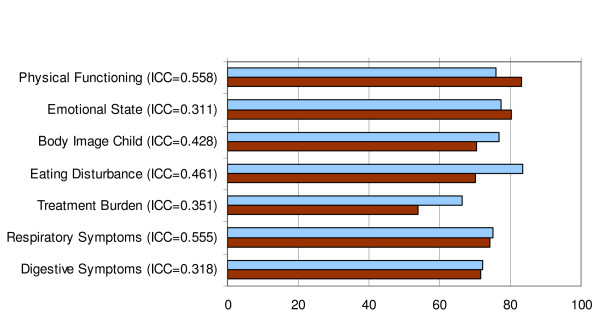
**Convergent validity of the German CFQ-R, Child and Parent versions**. The blue bars represent mean values of the CFQ-R Child version, the brown bars represent those of the CFQ-R Parent version. ICC values show convergence between self- and proxy rating.

Figure 1 shows the mean rating of children and parents in the dimensions covered congruently by both questionnaires. Correlations between self- and proxy rating are higher in "Physical Functioning" and "Respiratory Symptoms" (ICC = 0.56), but lower in all the other dimensions (ICC = 0.31 - 0.46).

## Discussion

The purpose of this study is to evaluate the psychometric properties of the German CFQ-R, Child and Parent versions, a combined self- and proxy rating system for assessing HRQoL in schoolchildren with CF. The development and translation of the questionnaire are a way of delivering an objective and multi-lingual instrument that includes the CF-specific dimensions.

### Reliability

Internal consistency is very good in the CFQ-R, Parent version. In the Child version, three dimensions show lack of internal consistency ("Body Image" α = 0.57, "Social Limitations" α = 0.23, "Treatment Burden" α = 0.24). For "Body Image", the lack of consistency is negligible. "Treatment Burden" is a dimension with only three items, and for the German version no item deletion would increase α. The other dimension, "Social Limitations", has seven items focusing on different aspects of social life. The US CFQ-R, Child version, showed low internal consistency in the same three dimensions: "Treatment Burden" (α = 0.44), "Social" (α = 0.60) and "Body Image" (α = 0.60) [17].

The reproducibility of the German CFQ-R is very good in all dimensions of the Parent version and in all but one dimension of the Child version. It is satisfactory for "Digestive Symptoms" in the Child version. As this dimension has only one item, and as abdominal pain can be sporadic in children even in stable health conditions, very good reproducibility cannot be expected.

In the two dimensions with low internal consistency in the Child version of the CFQ-R, reproducibility is high ("Social Limitations" ICC = 0.65; "Treatment Burden" ICC = 0.61). Thus, all dimensions show high reliability in at least one of the two investigated coefficients which justifies maintaining the structure.

### Construct Validity

The majority of item loadings support the model for both German questionnaires. For the US version, multitrait analyses were performed which also showed adequate item-internal and item-discriminant validity for the majority of items [17]. The number of item loadings not showing ideal values in the German version is acceptable. The more so as the importance of factor analysis has been critically discussed by several authors [33,34]. Each item seems to be relevant to the content. This content and its meaningfulness to the patient, the face validity, can be considered more important than a mathematical linkage.

With respect to content and form, the structure of the CFQ-R is not changed, which also ensures the comparability of the international questionnaires to a large extent.

### Known Group Validity

The discriminatory property of the questionnaire with regard to known groups is good. It is not surprising that only few differences between children with a different disease status are found in this sample of relatively healthy patients. All significant differences show a higher HRQoL in children with mild disease compared to those with moderate/severe disease. In the US trial, no associations were found between pulmonary function and CFQ-R scores. However, results cannot be directly compared, since the US trial used Pearson product moment correlation [17].

The differences in the self-rating of boys and girls are interesting since no significant differences are found between either the parent rating or the objective health state for boys and girls. We interpret this as a different perception of the disease by the genders. Lower HRQoL in girls has been described earlier [35] and psychological impairments leading to lower HRQoL might be more frequent in girls. This has been shown in adolescent patients with CF [36]. Modi et al. found lower scores in female patients of school age exclusively on the Respiratory scale by using the T-Test [17]. Thomas et al. found a lower rating in the Emotional scale of the CFQ-R in female Australian children [37].

Female patients with CF have a worse outcome than male patients; several potential reasons for this "gender gap" are currently discussed [38-43]. The observed lower HRQoL of girls in childhood might be one of the indicators or a potential cause of the gender gap and should therefore be followed up.

### Convergent Validity

The convergent validity of the Child and Parent versions shows different degrees of correlation, with a higher correlation in dimensions that are easier for parents to observe, e.g. "Physical Functioning" and "Respiratory Symptoms", and lower correlation in other dimensions, e.g. "Emotional State". These results show that the perceptions of the child and the parent can differ. Thus, although self-rating in childhood is difficult, it facilitates access to information that cannot be achieved by proxy rating only. It was shown earlier in the literature that children and parents have different perceptions of the child's HRQoL. The information from self- and proxy rating should be seen as complementary rather than contradictory information [44].

In contrast to our results, in the US significant agreement was found only in the dimensions "Body Image", "Eating", "Treatment Burden", "Digestion", and "Respiratory" scales. Correlations were between 0.22 and 0.37. Parents overestimated the "Physical Wellbeing" of their child, but underestimated the HRQoL on the "Respiratory" scale [17].

Havermans et al. investigated the degree of agreement between children with CF and their parents in Belgium and found the highest agreement in "Eating disturbances" (ICC = 0.75) and moderate agreement in "Respiratory Symptoms" (ICC = 0.50), "Digestive Symptoms" (ICC = 0.41) and "Body Image" (ICC = 0.4). As expected, "Emotional Functioning" (identical to "Emotional State" in the German and the US version) and "Treatment Burden" showed a very low correlation. Surprisingly, "Physical Symptoms" (identical to "Physical Wellbeing") showed no significant correlation in this population [45].

Elkins et al. used the CFQ-R to evaluate differences in HRQoL between children aged 6 to 13 in a controlled trial of long-term inhaled hypertonic saline in Australia, assessing proxy-rating only. Here, a better HRQoL was observed in the intervention group in one dimension of the Parent version, the dimension "Digestion" [46], which does not seem to be explained by the investigated drug. More differences in the HRQoL between patients of the intervention and control groups might have been observed if the investigators had used the CFQ-R, Child version in addition.

The findings of the current study underline the importance of combining self- and proxy rating, not only in Germany.

### Strengths and limitations of questionnaire and study design

The CFQ-R stands out due to the unique child module which allows the assessment of HRQoL already at the age of 6. It combines self- and proxy rating and provides access to the subjective perception of the child. The use of all CFQ-R versions also makes it possible to compare HRQoL at the different stages of life and in this way to explore the impact of CF in the course of a lifetime.

In comparison to other studies investigating HRQoL in children with CF, the number of patients in this multi-centre study is very high. However, a higher number of patients would have increased the power for factor analysis, where inclusion of at least 10 subjects per item is recommended. The test-retest reliability can only be evaluated in one of the participating centers and only in children with stable condition whose families agreed to a repeated administration of the questionnaires. Here, percentage of children with good lung function and nutritional state is lower than in the whole study population and the Benchmarking population, which cannot be definitely explained. One possible explanation is that patients who are in a bad physical condition are more likely to participate even in a repeated administration of the questionnaires.

The number of patients in both samples is limited, which might influence the results and should be noted in the interpretation. Some other methodological limitations must be noted: The quality of data might be limited due to different interviewers and settings and due to missing data on health state in 24 of the 136 children. Unfortunately, information on socioeconomic status cannot be obtained for all patients. Only one child with severe disease participates in this study, so group comparison is limited to two instead of three disease-severity groups. This is not surprising, since in most cases modern therapies facilitate a good health state in childhood. The minimal clinically important difference (MCID) for the dimensions of HRQoL has so far only been found for the "Respiratory" scale of the US version, it is 4.0 points for patients with CF who are cronically infected with Pseudomonas aeruginosa and have stable respiratory symptoms [47]. Responsiveness to change is not evaluated in this study, but the observed differences between the known groups show that the measures are likely to be sensitive to changes in health state.

## Conclusion

Overall, the psychometric properties of the Revised German CFQ-R, Child and Parent versions, can be classified as good and comparable to those of the US version. Only a few limitations in the psychometric properties have to be noted. Internal consistency is problematic in two dimensions of the Child version, both dimensions have good reproducibility. Previous studies in other countries also showed problematic internal consistency in child questionnaires. It should be underlined that reproducibility and known-group validity show very good results for both, the Child and Parent versions of the German CFQ-R. The questionnaires are reliable and valid disease-specific HRQoL measures that allow discrimination between known groups, indicating that the questionnaires are sensitive to change. Differences between genders in perception of HRQoL need further investigation.

Both self-rating and proxy rating provide important complementary sources of information, and these should be assessed in combination. Use of the CFQ-R in clinical studies and in the German registry will soon provide data to evaluate sensitivity to change more profoundly and to determine the minimal clinically important difference.

The data presented in this article allow the use of the German CFQ-R, Child and Parent versions as a secondary endpoint, for clinical purposes as well as for national and international studies. In the future, when sensitivity to change is evaluated more profoundly, they should also be used as a primary outcome parameter.

The questionnaires and scoring manual are available free of charge from the authors.

## Competing interests

The authors declare that they have no competing interests.

## Authors' contributions

AS carried of the study, collected and analysed the data, and drafted the manuscript. KW advised for statistical analysis. NN supplied a part of the data assessed in the Benchmarking Project. UW approved the concept of the study and provided the infrastructure. DS conceived the study and supervised data analysis. All authors read and approved the final manuscript.

## Supplementary Material

Additional file 1 **Appendix 1**. Dimensions and English translation of items of the German CFQ-R Child Version Click here for file

Additional file 2 **Appendix 2**. Dimensions and English translation of items of the German CFQ-R Parent Version Click here for file

Additional file 3 **Table S6**. Factor analysis, German CFQ-R, Child version Click here for file

Additional file 4 **Table S7**. Factor analysis, German CFQ-R, Parent version Click here for file

Additional file 5 **Table S8**. CFQ-R Child version - Total Variance Explained by the Factors Click here for file

Additional file 6 **Table S9**. CFQ-R Parent version - Total Variance Explained by the Factors Click here for file
