# Supplementary material for: Health-related quality of life in children with cystic fibrosis: validation of the German CFQ-R
Source: Health Qual Life Outcomes. 2009 Dec 2;7:97. doi: 10.1186/1477-7525-7-97 (PMC2794264; doi:10.1186/1477-7525-7-97)
Supplement: Additional file 1 — Appendix 1. Dimensions and English translation of items of the German CFQ-R Child Version [file 1477-7525-7-97-S1.DOC]

Appendix 1:

Dimensions and English translation of items of the German CFQ-R Child Version

*Physical Wellbeing*

01 to walk as fast as others

02 to climb stairs as fast as others

03 to run, jump and climb

04 to run as quickly and as long as others

05 to be able to participate in sports

06 difficulty carrying or liftig heavy things

*Emotional Wellbeing*

1. to feel tired

08 to feel mad

09 to feel grouchy

10 to feel worried

11 to feel sad

12 trouble falling asleep

13 bad dreams

14 to feel good about oneself

*Social Limitations*

20 to get together with friends

21 to stay at home than wanted

22 to sleep away from home

23 to feel left out

24 often invited friends

25 to be teased by other children

26 to feel comfortable discussing illness with others

*Body Image*

27 to think you were too short

28 to think you were too thin

29 to think you were physically different from others

*Eating Disturbance*

15 to have trouble eating

17 to be pushed to eat

19 to enjoy eating

*Treatment Burden*

16 to stop playing for treatment

18 to be able to do all the treatments

30 to be bothered by treatments

*Respiratory Symptoms*

31 to cough during the day

32 to wake up during the night because of the cough

33 to cough up mucus

34 to have trouble breathing

*Digestive Symptoms*

35 stomach hurt
