# Supplementary material for: Health-related quality of life in children with cystic fibrosis: validation of the German CFQ-R
Source: Health Qual Life Outcomes. 2009 Dec 2;7:97. doi: 10.1186/1477-7525-7-97 (PMC2794264; doi:10.1186/1477-7525-7-97)
Supplement: Additional file 2 — Appendix 2. Dimensions and English translation of items of the German CFQ-R Parent Version [file 1477-7525-7-97-S2.DOC]

Appendix 2:

Dimensions and English translation of items of the German CFQ-R Parent Version

*Physical Wellbeing*

01 physical activities

02 to walk fast

03 to climb stairs

04 to carry heavy objects

05 to climb several flights of stairs

13 to be absent or late for school

14 participation in sports

15 difficulty walking

16 to recover after physical effort

*Energy*

08 to seem tired

09 to seem short-tempered

10 to seem in good condition

11 to seem grouchy

12 to seem energetic

*Emotional Wellbeing*

06 to seem happy

07 to seem worried

23 to seem withdrawn

25 to have less fun

26 to have trouble with others

*School Performance*

27 to have trouble concentrating

28 to keep up with school work

29 performance at school

*Body Image*

19 to feel small

20 to feel physically different from others

21 to feel too thin

*Eating Disturbance*

17 mealtimes are a struggle

44 to be persuaded to eat

*Treatment Burden*

18 treatments in way of activities

30 time-consuming therapies

31 to have difficulties with treatment

*Subjective Health Perception*

22 to feel healthy

24 to live a normal life

32 rating of health state

*Weight Problems*

33 to have trouble gaining weight

*Respiratory Symptoms**

34 congested airways

35 cough during day

36 mucus

38 weezing

39 to have trouble breething

40 to wake up at night due to cough

*Digestive Symptoms*

41 flatulences

42 diarrhoea

43 abdominal pain

*Item no. 37 delivers additional information on the quality of mucus, its value is not calculated within the score of HRQoL.
