# Supplementary material for: Health-related quality of life in children with cystic fibrosis: validation of the German CFQ-R
Source: Health Qual Life Outcomes. 2009 Dec 2;7:97. doi: 10.1186/1477-7525-7-97 (PMC2794264; doi:10.1186/1477-7525-7-97)
Supplement: Additional file 3 — Table S6. Factor analysis, German CFQ-R, Child version [file 1477-7525-7-97-S3.DOC]

Table 6: Factor analysis, German CFQ-R, Child version

| **Item no.** | **PHY** | **EMO** | **EAT** | **TREAT** | **SOC** | **BOD** | **RES** | **DIG** |
| --- | --- | --- | --- | --- | --- | --- | --- | --- |
| 01 | **0.77** | 0.17 | 0.13 | -0.04 | 0.02 | 0.15 | 0.16 | 0.06 |
| 02 | **0.68** | 0.17 | 0.14 | -0.02 | -0.15 | 0.09 | 0.37 | -0.05 |
| 03 | **0.80** | 0.14 | 0.29 | 0.07 | 0.11 | 0.20 | 0.26 | 0.06 |
| 04 | **0.78** | 0.14 | 0.11 | 0.05 | -0.04 | 0.25 | 0.16 | 0.04 |
| 05 | **0.67** | 0.05 | 0.12 | 0.18 | 0.28 | 0.05 | 0.12 | 0.18 |
| 06 | **0.21** | 0.14 | 0.02 | -0.02 | 0.03 | 0.66* | 0.17 | 0.10 |
| 07 | 0.54* | **0.22** | 0.24 | 0.05 | 0.01 | 0.23 | -0.04 | 0.16 |
| 08 | 0.25 | **0.49** | 0.09 | 0.04 | 0.09 | 0.34 | -0.00 | 0.37 |
| 09 | 0.15 | **0.55** | 0.28 | 0.09 | -0.07 | 0.41 | -0.14 | 0.20 |
| 10 | -0.00 | **0.69** | -0.00 | -0.10 | -0.04 | 0.04 | 0.19 | 0.03 |
| 11 | 0.20 | **0.73** | 0.01 | 0.05 | 0.09 | 0.29 | 0.10 | 0.07 |
| 12 | 0.10 | **0.12** | 0.05 | 0.07 | 0.10 | 0.08 | -0.00 | 0.76* |
| 13 | 0.06 | **0.57** | 0.08 | -0.04 | -0.04 | 0.28 | 0.51 | 0.25 |
| 14 | 0.05 | **0.52** | -0.07 | -0.19 | 0.56 | 0.01 | 0.21 | -0.12 |
| 15 | 0.16 | 0.07 | **0.70** | 0.08 | 0.12 | 0.17 | 0.10 | 0.10 |
| 17 | 0.09 | 0.14 | **0.74** | -0.17 | -0.00 | 0.15 | 0.26 | -0.11 |
| 19 | 0.25 | -0.08 | **0.71** | 0.16 | 0.04 | -0.18 | 0.03 | 0.05 |
| 16 | 0.06 | 0.14 | 0.29 | **-0.40** | 0.18 | 0.37 | 0.45 | -0.25 |
| 18 | 0.48 | 0.21 | 0.31 | **-0.30** | 0.08 | -0.05 | -0.05 | 0.14 |
| 30 | -0.09 | 0.29 | 0.27 | **-0.48** | 0.26 | 0.13 | -0.22 | -0.44 |
| 20 | 0.03 | 0.15 | -0.02 | -0.01 | **0.64** | -0.10 | 0.13 | -0.02 |
| 21 | -0.07 | -0.01 | 0.13 | -0.23 | **-0.14** | 0.45* | 0.17 | -0.15 |
| 22 | 0.01 | 0.04 | 0.03 | 0.71* | **0.16** | -0.12 | -0.14 | 0.05 |
| 23 | 0.21 | 0.44 | 0.20 | -0.01 | **-0.12** | 0.59* | 0.25 | 0.43 |
| 24 | 0.21 | -0.12 | 0.11 | 0.34 | **0.59** | 0.09 | -0.24 | 0.21 |
| 25 | 0.07 | 0.56* | 0.18 | -0.16 | **0.09** | 0.07 | 0.36 | 0.34 |
| 26 | -0.08 | -0.22 | 0.08 | -0.01 | **0.40** | -0.09 | -0.12 | 0.24 |
| 27 | 0.18 | 0.09 | 0.57* | -0.09 | -0.11 | **0.30** | 0.04 | 0.01 |
| 28 | 0.07 | 0.45 | 0.38 | 0.33 | -0.26 | **0.45** | 0.32 | -0.14 |
| 29 | 0.14 | 0.47 | 0.20 | 0.07 | -0.18 | **0.65** | 0.28 | -0.02 |
| 31 | 0.41 | 0.12 | 0.11 | 0.09 | -0.01 | 0.36 | **0.52** | 0.05 |
| 32 | 0.16 | 0.29 | 0.08 | -0.21 | 0.16 | 0.26 | **0.61** | -0.34 |
| 33 | 0.17 | 0.09 | 0.18 | -0.06 | -0.06 | 0.15 | **0.54** | 0.08 |
| 34 | 0.19 | 0.36 | 0.21 | 0.03 | 0.00 | 0.22 | **0.67** | 0.05 |
| 35 | 0.18 | 0.63* | 0.10 | 0.09 | 0.06 | 0.12 | 0.28 | **-0.15** |
|  |  |  |  |  |  |  |  |  |
|  |  |  |  |  |  |  |  |  |
| Extraction method: principal component analysis.  Rotation method: Promax with Kaiser normalization. | | | | | | | | |
|  |  |  |  |  |  |  |  |  |
| **Loadings to related factor in bold** | | | | | | | | |
| * Loadings to unrelated factor greater than loadings to related factor | | | | | | | |  |
| PHY Physical Functioning; EMO Emotional State; EAT Eating Disturbance; TREAT Treatment Burden; | | | | | | | | |
| SOC Social Limitations; BOD Body Image; RES Respiratory Symptoms; DIG Digestive Symptoms | | | | | | | | |
|  | | | | | | | | |
