# Supplementary material for: Health-related quality of life in children with cystic fibrosis: validation of the German CFQ-R
Source: Health Qual Life Outcomes. 2009 Dec 2;7:97. doi: 10.1186/1477-7525-7-97 (PMC2794264; doi:10.1186/1477-7525-7-97)
Supplement: Additional file 4 — Table S7. Factor analysis, German CFQ-R, Parent version [file 1477-7525-7-97-S4.DOC]

Table 7: Factor analysis, German CFQ-R, Parent version

| **Item No.** | **PHY** | **EMO** | **ENE** | **BOD** | **HEALTH** | **SCHOOL** | **TREAT** | **WEIGHT** | **RES** | **DIG** | **EAT** |
| --- | --- | --- | --- | --- | --- | --- | --- | --- | --- | --- | --- |
| 01 | **0.78** | 0.18 | 0.49 | 0.36 | 0.07 | 0.31 | 0.01 | 0.45 | 0.44 | 0.01 | 0.17 |
| 02 | **0.87** | 0.20 | 0.44 | 0.36 | 0.01 | 0.41 | 0.28 | 0.25 | 0.46 | 0.20 | 0.41 |
| 03 | **0.91** | 0.26 | 0.41 | 0.40 | 0.15 | 0.27 | 0.18 | 0.28 | 0.48 | 0.10 | 0.24 |
| 04 | **0.82** | 0.16 | 0.29 | 0.37 | 0.26 | 0.35 | 0.21 | 0.28 | 0.37 | 0.08 | 0.39 |
| 05 | **0.90** | 0.31 | 0.37 | 0.34 | 0.13 | 0.31 | 0.12 | 0.25 | 0.50 | 0.05 | 0.23 |
| 13 | **0.15** | 0.13 | 0.16 | 0.20 | -0.03 | 0.28 | 0.01 | 0.74* | 0.33 | 0.19 | 0.13 |
| 14 | **0.37** | 0.09 | 0.27 | 0.16 | 0.03 | 0.12 | 0.15 | 0.79* | 0.22 | -0.03 | 0.23 |
| 15 | **0.81** | 0.27 | 0.51 | 0.38 | 0.28 | 0.47 | 0.28 | 0.47 | 0.52 | 0.06 | 0.25 |
| 16 | **0.74** | 0.36 | 0.62 | 0.40 | 0.25 | 0.44 | 0.00 | 0.38 | 0.46 | 0.11 | 0.27 |
| 06 | 0.24 | **0.33** | 0.75* | 0.15 | 0.19 | 0.26 | 0.02 | 0.11 | 0.17 | 0.02 | 0.06 |
| 07 | 0.28 | **0.04** | 0.21 | 0.45 | 0.48* | 0.40 | 0.09 | 0.43 | 0.31 | -0.04 | 0.15 |
| 23 | 0.34 | **0.66** | 0.43 | 0.26 | 0.08 | 0.16 | 0.24 | 0.22 | 0.32 | 0.04 | 0.20 |
| 25 | 0.43 | **0.69** | 0.39 | 0.29 | 0.09 | 0.35 | -0.05 | 0.22 | 0.16 | 0.13 | 0.19 |
| 26 | 0.20 | **0.83** | 0.21 | 0.27 | 0.31 | 0.33 | 0.05 | 0.14 | 0.18 | 0.11 | 0.18 |
| 08 | 0.44 | 0.17 | **0.67** | 0.20 | 0.04 | 0.21 | 0.28 | 0.19 | 0.25 | 0.13 | 0.26 |
| 09 | 0.27 | 0.19 | **0.14** | 0.06 | 0.77* | 0.18 | 0.18 | 0.06 | 0.07 | 0.20 | 0.13 |
| 10 | 0.50 | 0.38 | **0.65** | 0.55 | 0.34 | 0.41 | 0.00 | 0.51 | 0.52 | 0.08 | 0.28 |
| 11 | 0.23 | 0.25 | **0.37** | 0.28 | 0.70* | 0.28 | 0.15 | 0.09 | 0.17 | 0.08 | 0.23 |
| 12 | 0.40 | 0.11 | **0.84** | 0.34 | 0.16 | 0.20 | -0.02 | 0.36 | 0.38 | 0.00 | 0.16 |
| 19 | 0.19 | 0.10 | 0.12 | **0.77** | 0.03 | 0.16 | 0.14 | 0.11 | 0.15 | 0.15 | 0.21 |
| 20 | 0.45 | 0.23 | 0.40 | **0.84** | 0.22 | 0.29 | 0.20 | 0.28 | 0.26 | 0.20 | 0.25 |
| 21 | 0.24 | 0.25 | 0.22 | **0.76** | 0.12 | 0.29 | 0.06 | 0.26 | 0.27 | 0.40 | 0.47 |
| 22 | 0.54# | 0.10 | 0.39 | 0.63# | **0.04** | 0.50# | -0.03 | 0.24 | 0.43# | 0.03 | 0.23 |
| 24 | 0.39# | 0.17 | 0.25 | 0.54# | **0.15** | 0.67# | 0.10 | 0.20 | 0.47# | -0.01 | 0.22 |
| 32 | 0.61# | 0.19 | 0.46 | 0.48# | **-0.05** | 0.58# | 0.06 | 0.28 | 0.65# | -0.10 | 0.28 |
| 27 | 0.18 | 0.43 | 0.13 | 0.20 | 0.39 | **0.65** | 0.19 | 0.30 | 0.20 | 0.11 | 0.24 |
| 28 | 0.20 | 0.19 | 0.12 | 0.14 | 0.14 | **0.80** | 0.08 | 0.15 | 0.26 | 0.07 | 0.11 |
| 29 | 0.31 | 0.18 | 0.39 | 0.31 | 0.10 | **0.68** | 0.36 | 0.18 | 0.19 | 0.19 | 0.27 |
| 18 | 0.25 | 0.03 | 0.34 | 0.25 | 0.25 | 0.46 | **0.64** | 0.18 | 0.24 | 0.05 | 0.26 |
| 30 | 0.15 | 0.36 | -0.02 | 0.14 | 0.14 | 0.11 | **0.69** | 0.25 | 0.29 | -0.03 | 0.22 |
| 31 | 0.38 | 0.03 | 0.24 | 0.31 | 0.11 | 0.32 | **0.77** | 0.14 | 0.29 | 0.01 | 0.13 |
| 33 | 0.22 | 0.06 | 0.23 | 0.35 | 0.09 | 0.06 | 0.26 | **0.41** | 0.12 | 0.34 | 0.69* |
| 34 | 0.49 | 0.16 | 0.34 | 0.28 | 0.02 | 0.36 | 0.21 | 0.36 | **0.87** | 0.07 | 0.26 |
| 35 | 0.60 | 0.13 | 0.38 | 0.36 | -0.05 | 0.47 | 0.14 | 0.44 | **0.83** | 0.04 | 0.23 |
| 36 | 0.40 | 0.16 | 0.29 | 0.24 | 0.11 | 0.25 | 0.15 | 0.20 | **0.85** | 0.07 | 0.21 |
| 38 | 0.46 | 0.52 | 0.16 | 0.15 | -0.12 | 0.40 | 0.10 | 0.19 | **0.42** | 0.22 | 0.31 |
| 39 | 0.58 | 0.20 | 0.43 | 0.44 | -0.06 | 0.55 | 0.02 | 0.21 | **0.54** | 0.29 | 0.17 |
| 40 | 0.48 | 0.18 | 0.34 | 0.33 | -0.13 | 0.35 | 0.21 | 0.43 | **0.59** | 0.18 | 0.52 |
| 41 | 0.06 | 0.11 | 0.03 | 0.22 | 0.11 | 0.08 | -0.04 | 0.11 | 0.01 | **0.76** | 0.11 |
| 42 | 0.09 | 0.12 | 0.13 | 0.17 | -0.01 | 0.15 | 0.04 | 0.05 | 0.15 | **0.84** | 0.29 |
| 43 | 0.26 | 0.09 | 0.14 | 0.30 | 0.36 | 0.21 | -0.00 | 0.28 | 0.15 | **0.64** | 0.13 |
| 44 | 0.36 | 0.16 | 0.21 | 0.34 | 0.23 | 0.29 | 0.17 | 0.23 | 0.32 | 0.19 | **0.88** |
| 17 | 0.26 | 0.26 | 0.19 | 0.26 | 0.04 | 0.25 | 0.09 | 0.16 | 0.24 | 0.12 | **0.90** |
| Extraction method: principal component analysis. Rotation method: Promax with Kaiser normalization. | | | | | | | | | | | |
| Item 37 is not included in factor analysis, it delivers additional information about sputum colour. | | | | | | | | | | | |
| **Loadings to related factor in bold** | | | | | | | | | | | |
| * Loadings to unrelated factor greater than loadings to related factor | | | | | | | | | | | |
| # Items of the dimension have concordant loadings to several factors | | | | | | |  |  |  |  |  |
| PHY Physical Functioning; EMO Emotional State; ENE Energy; BOD Body Image; HEALTH Subjective Health Perception; | | | | | | | | | | | |
| SCHOOL School Performance; TREAT Treatment Burden; WEIGHT Weight Problems; RES Respiratory Symptoms; | | | | | | | | | | | |
| DIG Digestive Symptoms; EAT Eating Disturbance; | | | | | | | | | | | |
