# Supplementary material for: Health-related quality of life in children with cystic fibrosis: validation of the German CFQ-R
Source: Health Qual Life Outcomes. 2009 Dec 2;7:97. doi: 10.1186/1477-7525-7-97 (PMC2794264; doi:10.1186/1477-7525-7-97)
Supplement: Additional file 5 — Table S8. CFQ-R Child version - Total Variance Explained by the Factors [file 1477-7525-7-97-S5.DOC]

Table 8:

CFQ-R Child version - Total Variance Explained by the Factors

| **Dimensions of**  **Health-Related Quality of Life** | **CFQ-R Child version** | |
| --- | --- | --- |
|  | % of Variance | Cumulative % |
| Physical Functioning | 17 | 17 |
| Emotional State | 8 | 25 |
| Respiratory Symptoms | 7 | 32 |
| Eating Disturbance | 6 | 37 |
| Body Image | 5 | 42 |
| Social Limitations | 4 | 47 |
| Digestive Symptoms | 4 | 50 |
| Treatment Burden | 3 | 54 |
