# Supplementary material for: Health-related quality of life in children with cystic fibrosis: validation of the German CFQ-R
Source: Health Qual Life Outcomes. 2009 Dec 2;7:97. doi: 10.1186/1477-7525-7-97 (PMC2794264; doi:10.1186/1477-7525-7-97)
Supplement: Additional file 6 — Table S9. CFQ-R Parent version - Total Variance Explained by the Factors [file 1477-7525-7-97-S6.DOC]

Table 9:

CFQ-R Parent version - Total Variance Explained by the Factors

| **Dimensions of**  **Health-Related Quality of Life** | **CFQ-R Parent version** | |
| --- | --- | --- |
|  | % of Variance | Cumulative % |
| Physical Functioning | 28 | 28 |
| Respiratory Symptoms | 7 | 34 |
| Body Image | 5 | 40 |
| School Performance | 5 | 45 |
| Energy | 4 | 49 |
| Eating Disturbance | 4 | 53 |
| Emotional State | 4 | 57 |
| Digestive Symptoms | 4 | 60 |
| Treatment Burden | 3 | 63 |
| Subjective Health Perception | 3 | 66 |
| Weight Problems | 3 | 69 |
